# The Prevalent Use of Complementary and Alternative Medicine Among Patients With Chronic Disease in the Al-Madinah Population of Saudi Arabia

**DOI:** 10.7759/cureus.51130

**Published:** 2023-12-26

**Authors:** Ahmed S Metwally, Ibrahim A Atallah, Ibrahim K Almutairi, Mansour S Alzand, Mohammed S Alqabli

**Affiliations:** 1 Family and Community Medicine, Taibah University, Medina, SAU; 2 Family and Community Medicine, Suez Canal University, Ismailia, EGY; 3 Medicine, Taibah University, Medina, SAU

**Keywords:** saudi arabia, acupuncture, cupping, herbs, complementary and alternative medicine

## Abstract

Background: Global interest in complementary and alternative medicine (CAM) has recently risen, particularly in Saudi Arabia, and the use of CAM is gaining popularity as a healthcare option.

Objectives: This study aims to assess the prevalence of CAM use among patients with chronic diseases and identify the reasons for resorting to CAM in Al-Madinah, Saudi Arabia.

Methods: A cross-sectional study was conducted in Al-Madinah City. Data for analysis were derived from 416 participants with chronic diseases. The data were collected using a valid, structured online questionnaire that was designed to extract socio-demographic data as well as data on the predominant use of herps, hijama (cupping), acupuncture, and other specific CAM applications. The collected data were analyzed using appropriate statistical methods.

Results: Of the 416 participants, 164 (39.4%) were men, and 96.9% were Saudi citizens. Approximately one-third of the studied participants had diabetes (34.1%), while 29.3%, 25.2%, 15.1%, and 12.3% had hypertension, obesity, asthma, and gastrointestinal tract disease, respectively. The prevalence of herb and natural supplement use was 89.2%. The most commonly used herbs and natural supplements were ginger (55.5%), honey and its derivatives (53.4%), cinnamon (45.4%), and frankincense (33.4%). Of the studied participants, 36.1% and 6.5% reported undergoing cupping and acupuncture, respectively. No statistically significant differences were found between the use of herbs and acupuncture or any of the studied factors. Further, the use of acupuncture exhibited no significant differences. However, the use of cupping displayed statistically significant differences in age, sex, and income among the participants.

Conclusion: The prevalence of CAM use among patients with chronic diseases in Al-Madinah is high. It appears to play an essential role in health care, particularly in treating patients with chronic diseases in this population. Therefore, promoting research in the field of CAM is warranted.

## Introduction

Complementary and alternative medicine (CAM) is a different kind of medicine that can be used together with regular medicine, while alternative medicine is used instead of regular medicine [1]. It employs medical practices based on cultural beliefs and experiences that have neither been entirely integrated into the current healthcare system nor validated via multiple clinical studies on chronic diseases worldwide [2]. According to the WHO, over 75% of the world’s population relies on CAM, mainly herbs (medicinal plants), for health care [3]. It is still practiced, despite the notion that modern treatment is more effective. More than 70% of the population in developing countries continues to heavily rely on CAM [4].

As for the prevalence of CAM use in Saudi Arabia, around 46% of patients in healthcare facilities have used CAM at some point in their lifetime, and approximately 19% have been estimated to use CAM in the preceding 12 months [5]. In Saudi Arabia, approximately 69.9% of patients with cancer use CAM [6]. Its use has also been reported to be 29.9% and 26.1% in Lebanese and Congolese patients with hypertension, respectively [7,8], and 43.4% among Singaporean patients with cardiovascular disease [9]. The prevalence of CAM use in patients with diabetes in Saudi Arabia is reportedly 31.2% [10].

Previous studies have reported the common usage of CAM, such as herbs (including black seeds, honey, myrrh, and fenugreek), cupping (hijama), zamzam water, spiritual healing (Quran recitation), camel urine, and acupuncture, in Saudi Arabia [6,11-13]. The use of CAM in Saudi Arabia has not yet been fully elucidated, although perception is important for the successful counseling and effective support of the practice group. However, as few studies have explained how to use CAM, this study intends to enhance current knowledge regarding CAM applications in Al-Madinah, Saudi Arabia, to enable reasonable use. Therefore, this study aims to assess the prevalence of CAM use among patients with chronic diseases in Al-Madinah.

## Materials and methods

Study design and setting

This cross-sectional study was conducted using a self-administered questionnaire. The site of this research was Al-Madinah City, Saudi Arabia. According to the 2017 government statistics, the total population of the Al-Madinah region was approximately 1.2 million.

Study population and sampling

Individuals residing in Al-Madinah City were eligible to participate in this study. The sample size was calculated using the OpenEpi software based on the following assumptions: an anticipated frequency of 50%, a confidence level of 95%, and an alpha error of 5%. Accordingly, the calculated sample size was 384 participants.

Measurement tools

This study’s survey tool was a self-administered questionnaire that consisted of three parts. The first part comprised an informed consent form to be signed by each participant, while the second part surveyed sociodemographic data, such as sex, age, and income (adequate or inadequate). The third part of the questionnaire investigated the patient’s diagnosis (diabetes, hypertension, obesity, and/or cancer). After confirming their diagnoses, participants had to indicate what they predominantly used, i.e., herbs, hijama (cupping), and acupuncture, and if they used any other specific CAM resources. Moreover, their knowledge regarding CAM use was also interrogated. The questionnaires were distributed via social media applications (WhatsApp, Twitter, and Telegram). After having the survey’s purpose explained to them and subsequently agreeing to participate therein, participants completed the questionnaire. We included participants with chronic diseases and a history of CAM use and excluded those without chronic diseases.

Data collection tool and statistical analysis

A valid, structured online questionnaire (Google Forms, Alphabet Inc., Mountain View, CA, USA) was used to collect data (see Appendix A). Data analysis was performed using SPSS Statistics version 23 (IBM Corp., Armonk, NY, USA). Frequencies and percentages were used to display categorical variables. The chi-square test was used to evaluate the presence of an association between categorical variables. Statistical significance was set at p < 0.05.

Ethical consideration

The study was approved by the Research Committee of the College of Medicine, Taibah University (approval no. STU-21-031). The survey’s purpose was explained to the participants. Only after subsequently agreeing to participate were the participants allowed to complete the questionnaire. Also, data privacy and confidentiality were guaranteed.

## Results

**Figure 1:**
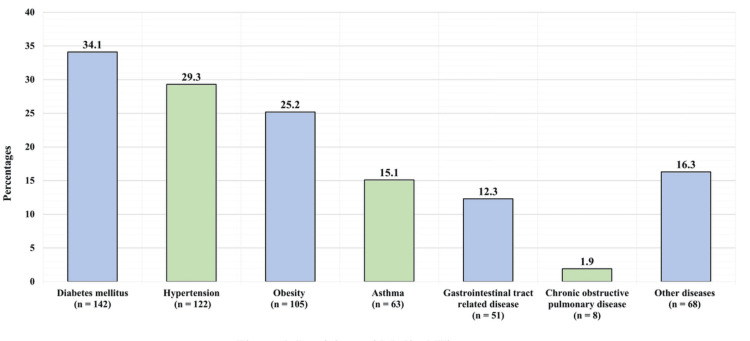
Medical history of participants

A total of 416 participants were included and analyzed in this study. Table 1 shows the participants’ sociodemographic profile. Of the participants, 129 (31%), 48 (11.5%), 104 (25%), and 135 (32.55%) were aged between 20 and 30, 31 and 40, 41 and 50, and >50 years, respectively. Regarding sex, 164 (39.4%) were men, and 252 (60.6%) were women. As regards nationality, 403 (96.9%) were Saudi, while 13 (3.1%) were not. In terms of education, 16 (3.8%), 57 (13.7%), and 343 (82.5%) had an intermediate school education or below, a high school education, and a university education, respectively. Pertaining to income, 304 (73.1%) reported earning sufficient income, while 112 (26.9%) reported earning insufficient income. Among the participants, 142 (34.1%) had diabetes mellitus, 122 (29.3%) had hypertension, 105 (25.2%) had obesity, 63 (15.1%) had asthma, 51 (12.3%) had gastrointestinal tract disease, eight (1.9%) had chronic obstructive pulmonary disease, and 68 (16.3%) had other diseases (Figure 1).

**Table 1:** Sociodemographic profile of the participants (n = 416)

Sociodemographic profile	n	%
Age		
20 to 30 years	129	31.00
31 to 40 years	48	11.50
41 to 50 years	104	25.00
Older than 50 years	135	32.50
Gender		
Male	164	39.40
Female	252	60.60
Nationality		
Saudi	403	96.90
Non-Saudi	13	3.10
Education		
Intermediate school education and less	16	3.80
High school education	57	13.70
University education	343	82.50
Income		
Sufficient	304	73.10
Insufficient	112	26.90

Table 2 displays the participants’ perceptions and sources of motivation toward using CAM, as well as their adherence to CAM use. Of the participants, 349 (83.9%) considered alternative medicine and supplements beneficial for medical treatment, while 67 (16.1%) did not. As regards those who recommended CAM to the participants, 182 (43.8%), 92 (22.1%), 76 (18.3%), 20 (4.8%), 15 (3.6%), and 42 (10%) reported receiving counsel from family, social media, friends, physicians, alternative medicine practitioners, and other sources, respectively. Regarding adherence to medication, 283 (68%) confirmed adherence to their medications, while 133 (32%) reported non-adherence.

**Table 2:** Perception toward alternative medicine, source of encouragement toward alternative medicine, and adherence to medications among the participants

Question & Response	n	%
Do you think alternative medicine and supplements used for medical treatment are beneficial?		
Yes	349	83.9
No	67	16.1
Who recommended alternative medicine treatment for you? More than one can be chosen.		
Family	182	43.80
Social media	92	22.10
Friends	76	18.30
Physician	20	4.80
Alternative medicine practitioners	15	3.60
Others	42	10.00
Are you adherent to taking your medications?		
Yes	283	68
No	133	32

**Figure 2:**
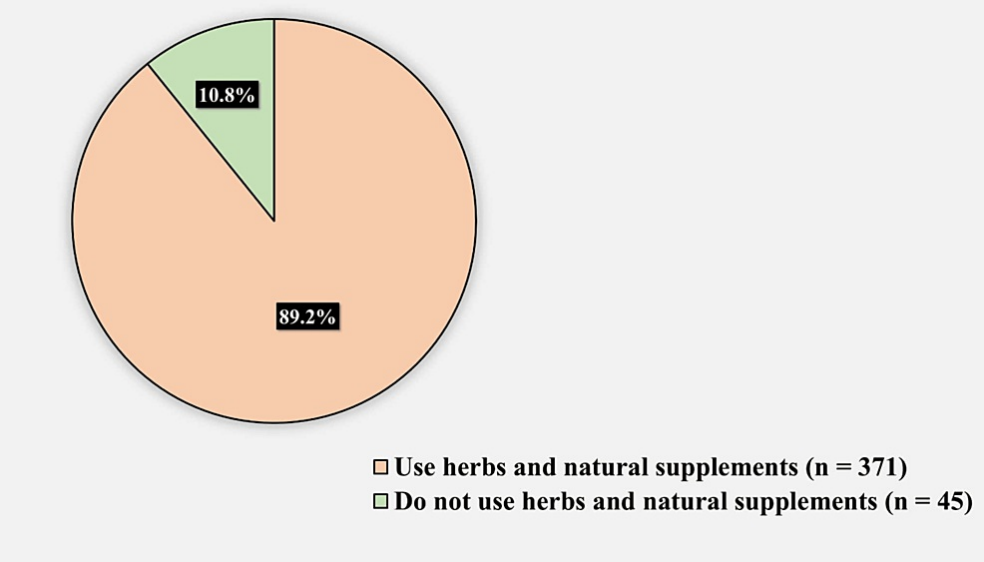
The prevalence of herbs and natural supplements use among participants

**Figure 3:**
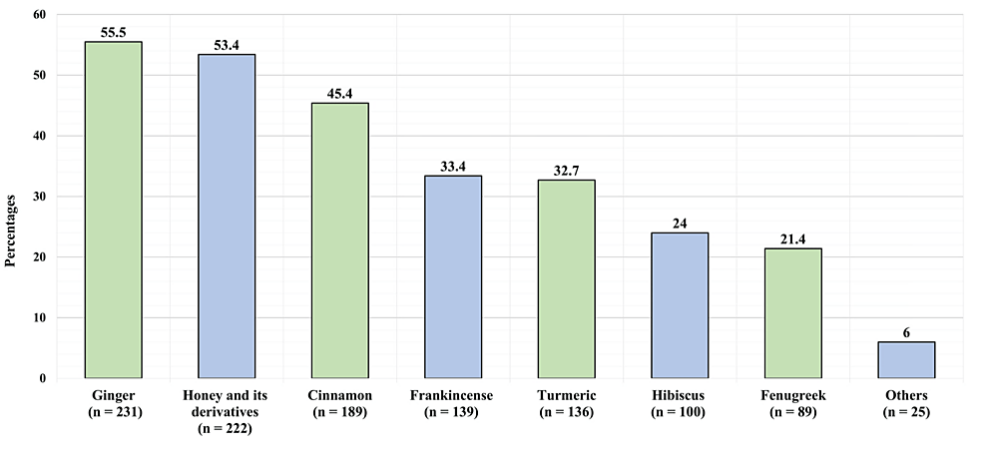
Herbs and natural supplements used by participants

Table 3 presents the factors associated with the prevalence of herb and natural supplement use. Age, sex, nationality, education, income, adherence to medication, history of acupuncture, and history of cupping were all non-significantly associated with the prevalence of herb and natural supplement use (Figure 2). The most commonly used herbs and natural supplements were ginger (231, 55.5%), honey and its derivatives used (222, 53.4%), cinnamon (189, 45.4%), and frankincense (139, 33.4%) (Figure 3).

**Table 3:** Factors associated with prevalence of herbs and supplement use *p-value is considered significant at  0.05 **p-value is considered significant at 0.001

Factors	Prevalence of herbs and supplement use		p-value
	Do not use	Use	
Age			
20 to 30 years	18 (14%)	111 (86%)	0.43
31 to 40 years	3 (6.3%)	45 (93.8%)	
41 to 50 years	12 (11.5%)	92 (88.5%)	
Older than 50 years	12 (8.9%)	123 (91.1%)	
Gender			
Male	19 (11.6%)	145 (88.4%)	0.68
Female	26 (10.3%)	226 (89.7%)	
Nationality			
Saudi	44 (10.9%)	359 (89.1%)	0.71
Non-Saudi	1 (7.7%)	12 (92.3%)	
Education			
Intermediate school education and less	2 (12.5%)	14 (87.5%)	0.60
High school education	4 (7%)	53 (93%)	
University education	39 (11.4%)	304 (88.6%)	
Income			
Sufficient	31 (10.2%)	273 (89.8%)	0.50
Insufficient	14 (12.5%)	98 (87.5%)	
Are you adherent to taking your medications?			
Yes	25 (8.8%)	258 (91.2%)	0.05*
No	20 (15%)	113 (85%)	
Have you ever undergone acupuncture?			
Yes	2 (7.4%)	25 (92.6%)	0.55
No	43 (11.1%)	346 (88.9%)	
Have you ever undergone cupping?			
Yes	16 (10.7%)	134(89.3%)	0.94
No	29 (10.9%)	237 (89.2%)	

**Figure 4:**
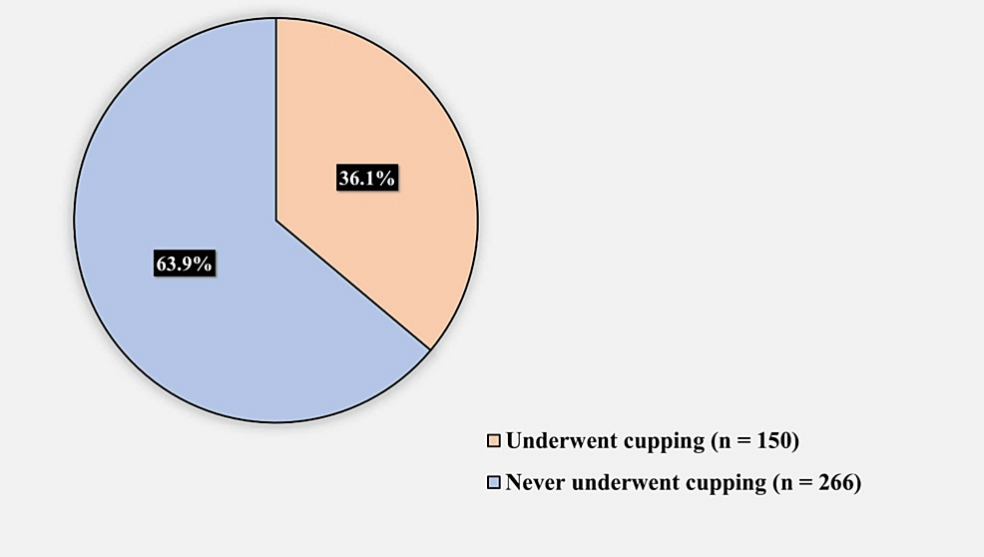
History of cupping among participants

Table 4 shows the factors associated with a history of cupping while Figure 4 shows the prevalence of cupping among participants. Age was significantly associated with cupping (p < 0.001); specifically, the older the age group, the higher the rate of cupping. Sex was also significantly associated with cupping (p = 0.039), where a higher rate of cupping was observed in men than in women (42.1% vs. 32.1%). Income was also significantly associated with cupping (p = 0.017); those with sufficient income exhibited a higher rate of cupping than those with insufficient income (39.5% vs. 26.8%). A history of acupuncture was also significantly associated with cupping (p = 0.001). In particular, those who had undergone acupuncture displayed a significantly higher rate of cupping than those who had not (66.7% vs. 33.9%). Sex, education, and adherence to medication were all non-significantly associated with cupping.

**Table 4:** Factors associated with a history of undergoing cupping *p-value is considered significant at  0.05 **p-value is considered significant at 0.001

Factors	History of undergoing cupping		p-value
	Yes	No	
Age			
20 to 30 years	27 (20.9%)	102 (79.1%)	0.001>**
31 to 40 years	16 (33.3%)	32 (66.7%)	
41 to 50 years	45 (43.3%)	59 (56.7%)	
Older than 50 years	62 (45.9%)	73 (54.15)	
Gender			
Male	69 (42.1%)	95 (57.9%)	0.03*
Female	81 (32.1%)	171 (67.9%)	
Nationality			
Saudi	147 (36.5%)	256 (63.5%)	0.32
Non-Saudi	3 (23.1%)	10 (76.9%)	
Education			
Intermediate school education and less	7 (43.8%)	9 (56.3%)	0.62
High school education	18 (31.6%)	39 (68.4%)	
University education	125 (36.4%)	218 (63.6%)	
Income			
Sufficient	120 (39.5%)	184 (60.5%)	0.17
Insufficient	30 (26.8%)	82 (73.2%)	
Are you adherent to taking your medications?			
Yes	108 (38.2%)	175 (61.8%)	0.19
No	42 (31.6%)	91 (68.4%)	
Have you ever undergone acupuncture?			
Yes	18 (66.7%)	9 (33.3%)	0.001**
No	132 (33.9%)	257 (66.1%)	

**Figure 5:**
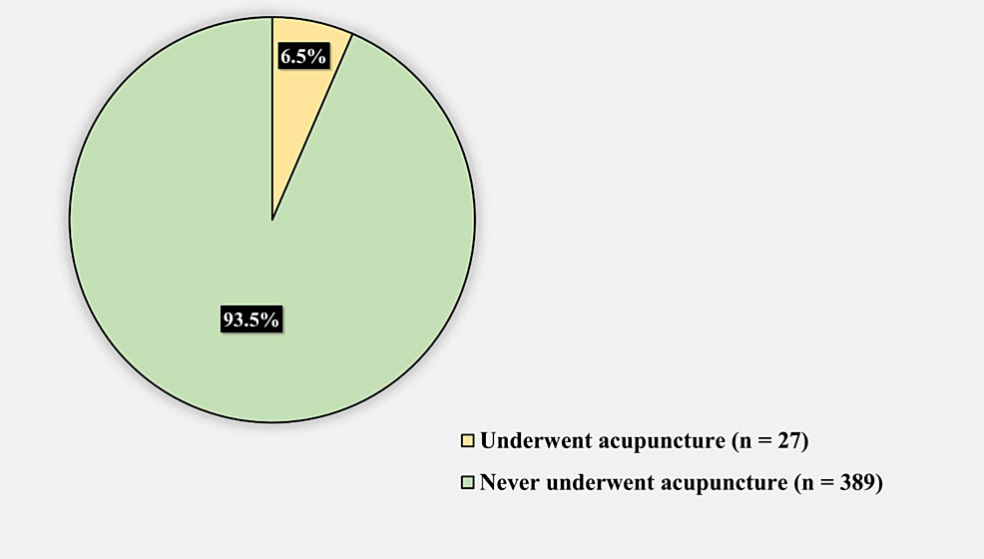
History of acupuncture among participants

Table 5 lists the factors associated with a history of acupuncture while Figure 5 captures the prevalence of acupuncture among participants. Age, sex, nationality, education, income, and adherence to medication were all non-significantly associated with acupuncture.

**Table 5:** Factors associated with a history of undergoing acupuncture *p-value is considered significant at  0.05 **p-value is considered significant at 0.001

Factors	History of undergoing acupuncture		p-value
	Yes	No	
Age			
20 to 30 years	7 (5.4%)	122 (94.6%)	0.39
31 to 40 years	4 (8.3%)	44 (91.7%)	
41 to 50 years	4 (3.8%)	100 (96.2%)	
Older than 50 years	12 (8.9%)	123 (91.1%)	
Gender			
Male	9 (5.5%)	155 (94.5%)	0.50
Female	18 (7.1%)	234 (92.9%)	
Nationality			
Saudi	26 (6.5%)	377 (93.5%)	0.85
Non-Saudi	1 (7.7%)	12 (92.3%)	
Education			
Intermediate school education and less	1 (6.3%)	15 (93.8%)	0.29
High school education	1 (1.8%)	56 (98.2%)	
University education	25 (7.3%)	318 (92.7%)	
Income			
Sufficient	20 (6.6%)	284 (93.4%)	0.90
Insufficient	7 (6.3%)	105 (93.8%)	
Are you adherent to taking your medications?			
Yes	19 (6.7%)	264 (93.3%)	0.78
No	8 (6%)	125 (94%)	

## Discussion

In the past few decades, CAM has gained popularity, possibly placing its users at risk. This is especially true considering the fact that CAM lacks systematic clinical trials and therefore evidence, casting doubt on its effectiveness and rendering its safety questionable [14]. This study aimed to estimate the prevalence of and explore the reasons behind CAM use among patients with chronic disease in Al-Madinah, Saudi Arabia.

This study’s prevalence of herb and natural supplement use among patients with chronic comorbidities was 371 (89.2%) participants, which exceeded that obtained by Al-Faris et al. (19.2% among citizens of Riyadh, Saudi Arabia), Al-Garni et al. (25% among patients with diabetes in Jeddah, Saudi Arabia), Jan et al. (30% among citizens of western Saudi Arabia), Al Saeedi et al. (30.1% among patients with diabetes in Mecca, Saudi Arabia), Al-Eidi et al. (30.4% among patients with diabetes in Riyadh), Abdo et al. (31.8% among patients with liver disease in Riyadh), Musaiger and Abahussain (37% among adolescents in Saudi Arabia), Lulebo et al. (42.5% among patients with hypertension in Kinshasa, Democratic Republic of the Congo), Al-Rowais et al. (43.2% among citizens of Riyadh), Sait et al. (54% among patients with cancer in Jeddah), Elolemy and Albedah (58.9% among citizens of Riyadh), Al Mansour et al. (60.9% among medical students in Al Majma’ah, Saudi Arabia), and Kamel et al. (64% among patients with diabetes in Jeddah) [5,8,11,13,15-23].

As suggested by Al-Faris et al., this high prevalence might have been attributed to patients’ beliefs regarding the nature of chronic illnesses, where they harbor false perceptions that chronic diseases can be cured by CAM, thereby blaming modern medicine for its inability to cure them and looking for alternatives [5]. Additionally, we assume that patients tend to use CAM owing to its affordability, extensive availability, and falsely presumed safety and benefits. Moreover, the possible side effects of modern medicinal drugs, the necessity to strictly adhere to a prescribed treatment plan, and the multiplicity of medications that need to be taken daily may drive patients with chronic diseases, especially older patients and those with disabilities, to explore alternatives. Finally, we suppose that our study yielded higher CAM-use rates than previous studies because we explored herb and natural supplement use as a single entity, whereas other studies explored them as separate entities.

Our study found a low-to-moderate prevalence of cupping in 150 (36.1%) participants. This finding corresponds with the results of AlGhamdi et al., Elolemy and Albedah, and Mohammad et al., wherein the prevalence of cupping was found to be 32%, 35.7%, and 45.4%, respectively [22,24,25]. As proposed by Alrowais and Alyousefi, this relatively low prevalence of cupping compared with that of other CAM practices is supposedly caused by the mandatory licensing of practitioners by the government as well as the strict sterilization, cleaning, and disinfection procedures required for cupping equipment, thus limiting its availability and prevalence [12]. We also assume that with increasing awareness in this social media era, people have grown to prefer blood donation over cupping, considering that the blood drawn during cupping is wasted, whereas that drawn during blood donation is used to save patients’ lives, an act that is extremely encouraged by the religion (Islam) practiced in the country under study. Notwithstanding, our result is considerably higher than that of Al-Faris et al. (2.1%) [5], Al-Rowais et al. (4.4%) [20], Jan et al. (9%) [16], and AlBedah et al. (13%) [26].

In a recent cross-sectional Saudi study on 386 patients in Riyadh, approximately 34.2% of the patients had previously undergone cupping therapy [27]. In general, the prevalence of cupping ranged from 2.1% to 70.6% across different settings in Saudi Arabia, including a range of 7.7% to 45.4% in various outpatient settings and 13% in a single primary care setting [5,20,26]. This discrepancy between the findings might have been related to the diverse methodologies used, sample sizes, and target populations. 

In Egypt, a total of 900 participants (750 from Ain Shams University Hospital and 150 from Benha University Hospital) were included in a cross-sectional study that assessed their practice of cupping. Of these participants, 68.1% had heard about cupping therapy, among whom 16% reported having undergone it. The predominant reason for undergoing cupping therapy was the treatment of bone and joint disorders [28].

In our study, the prevalence of acupuncture was extremely low (6.5%). Globally, it has been found to vary greatly. In the United Kingdom, 2.3 million traditional acupuncture treatments are performed annually. In the United States, the number of acupuncturists doubled between 2002 and 2012 [29]. In Australia, 9.5% and 6.2% of young and middle-aged women have consulted an acupuncturist, while 5.7% and 4.0% have used it, respectively. Regionally, however, our study findings are consistent with those of Mohammed et al., Al Bedahet al., and Elolemy and Albedah, who found acupuncture prevalence rates of 2% [25], 2.2% [26], and 9.8% [22], respectively. However, they contradict the results of AlGhamdi et al. and Al Mansour et al., who observed acupuncture prevalence rates of 18% [24] and 34.8% [23], respectively. We believe that acupuncture has the lowest prevalence compared with other CAM practices, mainly because it is invasive in nature and may cause pain if not performed by trained experts. Moreover, the origins and cultural background of acupuncture are rooted in China, not Saudi Arabia, thus rendering Saudis less receptive to this practice.

Among this study’s participants, 349 (83%) believe that CAM is beneficial. This finding supports that of Al-Faris et al. and Gad et al., who both found the perceived success of CAM to be a factor associated with CAM use [5]. We speculate that patients who use CAM develop a feeling of satisfaction premised on the fact that they would have spent time, effort, and possibly money to improve their health; such impressions are probably misinterpreted by patients as benefits of CAM rather than self-striving.

In this study, old age was significantly associated with cupping. This finding corroborates that of Al-Faris et al., who also found old age to be significantly associated with CAM use [5]. Further, in a recent Saudi study, cupping use was significantly more frequent in older individuals, men, and specific occupations [27]. Nevertheless, these findings contradict those of Lulebo et al., who found no significant association between age and the use of CAM [8]. We hypothesize that old people tend to be more religious than young people, making them more open to cupping therapy, especially considering the fact that cupping has an Islamic background and was encouraged by Prophet Mohammed.

In contrast to studies by Al-Faris et al., who found the female sex to be significantly associated with CAM use [5], and Lulebo et al., who reported no significant association between sex and CAM use [8], we found the male sex to be significantly associated with cupping. This is probably because cupping is not entirely painless and may leave bruises, rendering it a less preferred practice among women.

In correspondence with the study by Al-Faris et al., wherein high income was significantly associated with CAM use [5], we found high income to be significantly associated with cupping. We believe that this is an expected association, as the more affluent an individual becomes, the more receptive they become to new experiences.

In this study, having a history of acupuncture was significantly associated with cupping. This might have emanated from the fact that once a person is convinced that a benefit can be derived from CAM that results in them adopting a certain form of CAM, they are more likely to also practice other forms of CAM.

Strengths

To the best of our knowledge, this study is the first of its kind because it explores the prevalence rates of various CAM practices as well as the reasons patients with different chronic comorbidities engage in them. The study’s sample group comprised patients who presumably were on multiple medications and had comorbid cardiopulmonary conditions, both of which render such patients unsuitable for practices that lack evidence. This reveals the necessity of providing this patient subpopulation with appropriate knowledge and guidance and addressing the factors driving patients to seek alternatives to conventional medical treatment.

Limitations

This study had certain limitations that should be addressed by future studies. First, data were collected from participants via an online, self-administered survey, and this might have affected the accuracy of the results. Second, most of the participants were women (252, 60.6%), possibly causing bias in the results. Third, the study explored herbs and natural supplements as a single entity rather than exploring them individually, and this might have compromised the accuracy of the results. Finally, many of the factors previously reported to encourage CAM use, such as perceived failure of medical treatment, preference for natural substances, and long appointment intervals [5], were not explored in this study, thus potentially masking the actual reasons behind CAM use in some participants.

## Conclusions

This study examines the utilization of herb and natural supplements, cupping, and acupuncture among patients with chronic comorbidities. The findings reveal a notable prevalence of herb and natural supplement use, with a significant number of patients incorporating these therapies into their healthcare practices. Cupping was also found to be a common practice, particularly among older individuals, males, those with higher incomes, and those with a history of acupuncture.

Based on the study's findings, several key recommendations can be made. Firstly, there is a need to raise awareness about potential drug interactions associated with herb and natural supplement use. Implementing awareness campaigns can help educate individuals about the risks of drug interactions, some of which can have life-threatening consequences. Secondly, strict regulations should be imposed on cupping and acupuncture centers, as well as practitioners. These regulations are essential to address the potential transmission of infectious diseases when sterilization and disinfection practices are ineffective. Furthermore, healthcare providers in primary health centers should proactively inquire about patients' utilization of CAM and provide relevant consultation where necessary. This approach ensures that a patient's CAM usage is taken into consideration during their healthcare journey, allowing for a comprehensive and integrated approach to treatment.

Lastly, future researchers are encouraged to collect data through interviews, employ larger sample sizes, and explore the utilization of herbs and natural supplements separately. These methodological improvements aim to minimize bias and enhance the accuracy of research results. By conducting further research using robust methodologies, we can gain a deeper understanding of CAM utilization patterns and their impact on patient outcomes.

In summary, this study underscores the importance of healthcare providers being aware of patients' utilization of CAM therapies, including herb and natural supplements, cupping, and acupuncture. The recommendations put forth emphasize the significance of education, regulation, proactive patient engagement, and rigorous research methodologies to ensure the safe and effective integration of CAM therapies into healthcare practices.

## Appendices

Appendix A

**Table 6:** Questionnaire used to assess participants in this study

Questionnaire												
Q1. Do you agree to participate in this survey?												
Yes							No					
Q2. What is your gender?												
Male							Female					
Q3. What is your age?												
20 - 30 years		31 - 40 years				41 - 50 years		Older than 50 years				
Q3. What is your nationality?												
Saudi							Non-Saudi					
Q4. What is your educational level?												
Illiterate		Elementary School education				Middle School Education		High school Education			University education	
Q5. Is your income sufficient or insufficient?												
Sufficient							Insufficient					
Q6. Are you diagnosed with any chronic illnesses? Choose from the following.												
Diabetes	Hypertension				Obesity		Cancer		Chronic obstructive pulmonary disease			Other name it
Q7. Have you used alternatives and supplements to medical treatment? Choose from the following												
Cinnamon			the Garlic				Honey and its derivatives			Fenugreek		
Ginger			Hibiscus				Turmeric			Frankincense		
Q8. Have you ever undergone cupping?												
Yes							No					
Q9. Have you ever undergone acupuncture?												
Yes							No					
Q10. Have you undergone any other alternative medicine therapy? Name it.												
Q11. Who recommended alternative medicine treatment for you? More than one can be chosen.												
Family	Social media			Friends			Physician		Alternative medicine practitioners			Other name it
Q12. Do you think alternative medicine and supplements used for medical treatment are beneficial?												
Yes							No					
Q13. Are you adherent to taking your medications?												
Yes							No					
